# Synthesis of a gadolinium based-macrocyclic MRI contrast agent for effective cancer diagnosis

**DOI:** 10.1186/s40824-018-0127-9

**Published:** 2018-06-13

**Authors:** Yohan Jeong, Kun Na

**Affiliations:** Department of Biotechnology, The Catholic University of Korea, 43 Jibong-ro, Wonmi-gu, Bucheon-si, Gyeonggi do 420-743 South Korea

**Keywords:** Magnetic resonance imaging, DOTA, Contrast agent, Macrocyclic chelator, Nephrogenic systemic fibrosis

## Abstract

**Background:**

Gadolinium-based contrast agents are widely used as a contrast agent for magnetic resonance imaging. Since gadolinium ions are toxic, many chelators are developed to bind gadolinium ions to prevent free gadolinium-associated disease. However, many reports indicated that linear chelator-based contrast agents are associated with nephrogenic systemic fibrosis (NSF) in patients with low kidney function. Therefore, the demand for stable macrocyclic chelator-based contrast agent is now increasing.

**Method:**

1,4,7,10-Tetraazacyclododecane-1,4,7,10-tetraacetate (DOTA) was conjugated to lactobionic acid (LBA) through DCC-NHS coupling reaction. Gd^3+^ (gadolinium ion) was chelated to 1,4,7,10-Tetraazacyclododecane-1,4,7,10-tetraacetate-lactobionic acid (DOTA-LAE) and free Gd^3+^ was removed using a cation exchange column. In vitro cytotoxicity of contrast agent towards normal cells was measured using MTT assay. For in vivo MR imaging, contrast agents were intravenously injected to tumor-bearing mice and imaged by a MR imaging scanner.

**Results:**

This new macrocyclic gadolinium-based contrast agent showed enhanced in vitro paramagnetic properties compared to Gadovist. In addition, Gd-DOTA-LAE showed a 29% increased contrast enhancement of tumor tissue compared to normal tissue within 20 min past IV injection.

**Conclusions:**

We developed a new macrocyclic T1-weighted MR contrast agent. This new contrast agent offers various opportunities for cancer detection and diagnosis.

## Background

Early detection of cancer is essential for treatment and the survival rate of patients [1, 2]. Magnetic resonance imaging (MRI) is most frequently used imaging method for detection and diagnosis of cancer. MRI is a noninvasive method to detect soft tissue, such as organs, ligament, cartilage, and cancer regions without exposing to radiation. However, it is still hard to distinguish between tumor regions and normal region. Many contrast agents (CAs) have been developed to enhance contrast intensity and contrast effect on region of interest. Gadolinium, manganese, iron oxide, and iron platinum-based CAs are used for clinical application, because Gd^3+^-based T1 CAs have been proven its great safety in many clinical cases and Gd^3+^-based T1 CAs are the most widely applied CAs on these days. Moreover, there have been various attempts to improve the signal intensity and sensitivity of Gd^3+^-based CAs to region of interest [3–6]. Improvement of signal intensity is related to the concentration of CAs in region of interest and shortening the longitudinal relaxation time of surrounding water protons nearby Gd^3+^ ions [7–10]. One of the methods is accumulation of CAs using conjugation with bioactive moieties to increase concentration of CAs in specific regions, tumor specific antibody, and stimuli-responsive polymer [11–16]. The other method is decreasing longitudinal relaxation time of surrounding water proton using CAs [17, 18]. Increasing the signal intensity of the CAs is also important, but there are more concerns about stability of CAs. For a decade, there have been reports of nephrogenic systemic fibrosis (NSF) associated with the use of Gd^3+^-based CAs [19, 20], especially for patients with low kidney function [21]. NSF is a rare and serious disease that causes severe fibrosis of skin and internal organs [22]. It is known that release of free Gd^3+^ from unstable chelator may associated with NSF. For this reason, The European Medicine Agency (EMA) has recommended a restriction for linear chelator-based CAs to prevent against any dangers from release of Gd^3+^ [23]. Therefore, there are demands for stable and safe macrocyclic chelator CAs. The purpose of this study was to design new macrocyclic gadolinium-based contrast agent for MR imaging of tumors. In this respect, we designed a hydroxyl group rich material conjugated CA for high water proton exchange to improve T1-weighted signal intensity.

## Method

### Materials

Lactobionic acid, ethylenediamine, gadolinium chloride hexahydrate, trifluoroacetic acid(TFA), N, N-dicyclohexylcarbodiimide (DCC), N-hydroxysuccinimide (NHS), Chelex ®100 (100~ 200 mesh), 3-(4,5-Dimethyl-2-thiazolyl)-2,5-diphenyl-2H-tetrazolium bromide (MTT), and xylenol orange disodium salt were purchased from Sigma Aldrich (St. Louis, MO, USA). Tri-tert-butyl 1,4,7,10-Tetraazacyclododecane-1,4,7,10-tetraacetate (tri BOC-DOTA) was purchased from Tokyo Chemical Industry Corporation (Tokyo, Japan). N, N-dimethylformamide (DMF), dimethyl sulfoxide (DMSO) and hydrochloric acid (35–37%) were purchased from Junsei Chemical Co. Ltd. (Tokyo, Japan). Ether and acetone were purchased from Samchun Pure chemical Co. Ltd. (South Korea). Dimethyl sulfoxide-d6 (DMSO-d6) and deuterium oxide(D2O) were purchased from Cambridge Isotope Laboratories (Andover, MA, USA). Gadobutrol (Gadovist) was acquired from Bayer (Leverkusen, Germany). Chang cell (human epithelial liver cells) and HCT 116 cell (human colon carcinoma) were acquired from the American Type Culture Collection (ATCC CCL 13, USA).

### Synthesis of lactobionic acid-ethylenediamine (LAE)

DOTA-lactobionic acid was synthesized using carbodiimde reactions. Briefly, 1 g of lactobionic acid (LBA) was dissolved in DMF (10 mL) and activated by DCC (1.2 mol equiv. of LBA) and NHS (1.2 mol equiv. of LBA) at room temperature for 12 h. The by-product of the reaction, dicyclohexylurea, was removed by 0.45 μm syringe filter. Ethylenediamine (10 mol equiv. of LBA) was diluted with DMF (10 mL) and added dropwise to the solution of activated LBA. The solution reacted for 24 h at room temperature and purified by precipitation in cold ether and washed three times with ether. Lactobionate-ethylendiamine (LAE) was obtained under vacuum. The product of LAE was confirmed by 300 MHz ^1^H NMR spectrometer. (Bruker, Germany).

### Synthesis of DOTA-LAE

Five hundred seventy-three milligrams of Tri-tert-butyl 1,4,7,10-Tetraazacyclododecane-1,4,7,10-tetraacetate (tri BOC-DOTA) was dissolved in DMF (5 mL) and activated by DCC (1.2 mol equiv. of tri BOC-DOTA) and NHS (1.2 mol equiv. of tri BOC-DOTA) at room temperature for 12 h. The by-product of the reaction, dicyclohexylurea, was removed by 0.45 μm syringe filter. Four hundred eighty milligrams of LAE (1.2 mol equiv. of tri BOC-DOTA) was added into the solution and reacted for 24 h at room temperature. Product (tri BOC-DOTA-LAE) was precipitated by adding cold ether and kept at deep freezer for 10 mins to complete the precipitation. The tri BOC-DOTA-LAE was washed three times with ether then dried under vacuum. 0.5 g of tri BOC-DOTA-LAE was dissolved in 3 ml of 75% TFA in DCM and treated for 40 min to remove tert-butyl group and dried under reduced pressure. The final product was dissolved in distilled water (D.W) and placed in a dialysis bag (molecular weight cutoff 500 Da) against D.W for 3 days. DOTA-LAE was obtained by lyophilizing. The product of DOTA-LAE was confirmed by 300 MHz ^1^H NMR spectrometer. (Bruker, Germany).

### Grafting of gadolinium (III) to DOTA-LAE

Five hundred milligrams of DOTA-LAE was dissolved in D.W (10 ml) and gadolinium chloride hexahydrate (GdCl_3_∙H_2_0) (1.2 mol equiv. of DOTA-LAE) was added to this solution, and pH was adjusted to 6 with 0.1 M KOH solution [24]. The solution was heated to 40 °C for 24 h under stirring. Free Gd^3+^ ion was removed by dialysis (molecular weight cutoff 500 Da) in D.W for 3 days. One gram of chelax®100 resin was added to the solution at pH 5 and stirred gently for 1 h, then supernatant was decanted and lyophilized.

### Estimation of paramagnetic properties of Gd-DOTA-LBA

The T1 and T2 relaxation times of LBA and Gadovist were measured in test tube with various Gd^3+^ concentrations. The longitudinal rate (R_1_) and transverse rate (R_2_) were obtained by calculating the slope of the 1/T1 and 1/T2. All studies were performed on a 4.7 T animal MRI scanner (Biospec 47/40, Bruk-er BioSpin, Ettlingen, Germany) with 72 mm coil at Korea Basic Science Institute in Ochang.

### Cell culture

Chang cells and HCT 116 cells were cultured in Dulbecco’s Medium with Earle’s BSS (DMEM, Hyclone) supplemented with 10% (*v*/v) fetal bovine serum (FBS), containing100 IU /mL of penicillin, and 100 μg/mL of streptomycin at 37 °C under 5% CO_2_.

### Cytotoxicity studies of Gd-DOTA-LAE

The cytotoxicities of Gd-DOTA-LAE, Gadobutrol (Gadovist), and free Gd^3+^ were evaluated for 24 h using the (3-(4,5-Dimethyl-2-thiazolyl)-2,5-diphenyl-2H-tetrazolium bromide) MTT assay. Chang liver cells were seeded into 96 well plates and incubated under 5% CO_2_ at 37 °C. Gd-DOTA-LAE, Gadovist, and free Gd^3+^ in 100 μl of SF medium were added to each well in a concentration- and time-dependent manners. To measure cytotoxicities by time-dependent manners, 0.1 mM concentration of Gd-DOTA-LAE, Gadobutrol (Gadovist), and free Gd^3+^ were treated at each time point. Then 10 μl of MTT solution (0.5 mg/ml) was added to each well and incubated for additional 4 h. Media containing MTT was removed and the blue formazan crystals trapped in living cells were dissolved in DMSO (100 μl). The absorbance of formazan crystal in the wells was measured using a microplate reader at 570 nm.

### In vivo MR imaging

All procedures were approved by the Institutional Animal Care and Use Committee (IACUC) of the Catholic University of Korea in accordance with the “Principles of Laboratory Animal Care”, NIH publication no. 85-23, revised in 1985. HCT 116 cells (1.0 × 10^6^cells) were suspended in 100 μl of phosphate buffer saline and injected subcutaneously into male BALB/c mice. When the tumor volume reached about 300 mm^3^, GD-DOTA-LAE (0.1 mmol/kg) and Gadovist (0.1 mmol/kg) were injected into the lateral tail vein of male BALB/c mice. Mice were placed in a 4.7 T animal MRI scanner (Biospec 47/40, Bruker BioSpin, Ettlingen, Germany) and MR images were obtained before injection and every 5 mins for 1 h following application.

### Removal of free gadolinium ion

One gram of DOTA-LBE was dissolved in 100 ml of D.W and 5 g of chelex ®100 resin was added into the solution to remove unreacted Gadolinium. Solution was stirred gently for 1 h and supernatant was filtered and lyophilized. Concentration of free gadolinium ion was determined using xylenol orange method [25]. The ratio of absorbance at 573 and 433 nm is proportional to the free Gd^3+^ concentration. The chelated gadolinium (Gd^3+^) contents were estimated using Inductively coupled plasma-optical emission spectrometry (ICP-MS) (PerkineElmer, Optima 4300 DV, Norwalk, CT, USA).

### Statistical analysis

Data was represented as mean ± SD for all the groups. The statistical analysis was performed by Student’s t-test and *p* < 0.01 was considered statistically significant.

## Results

### Synthesis of Gd-DOTA-LAE

**Fig. 1:**
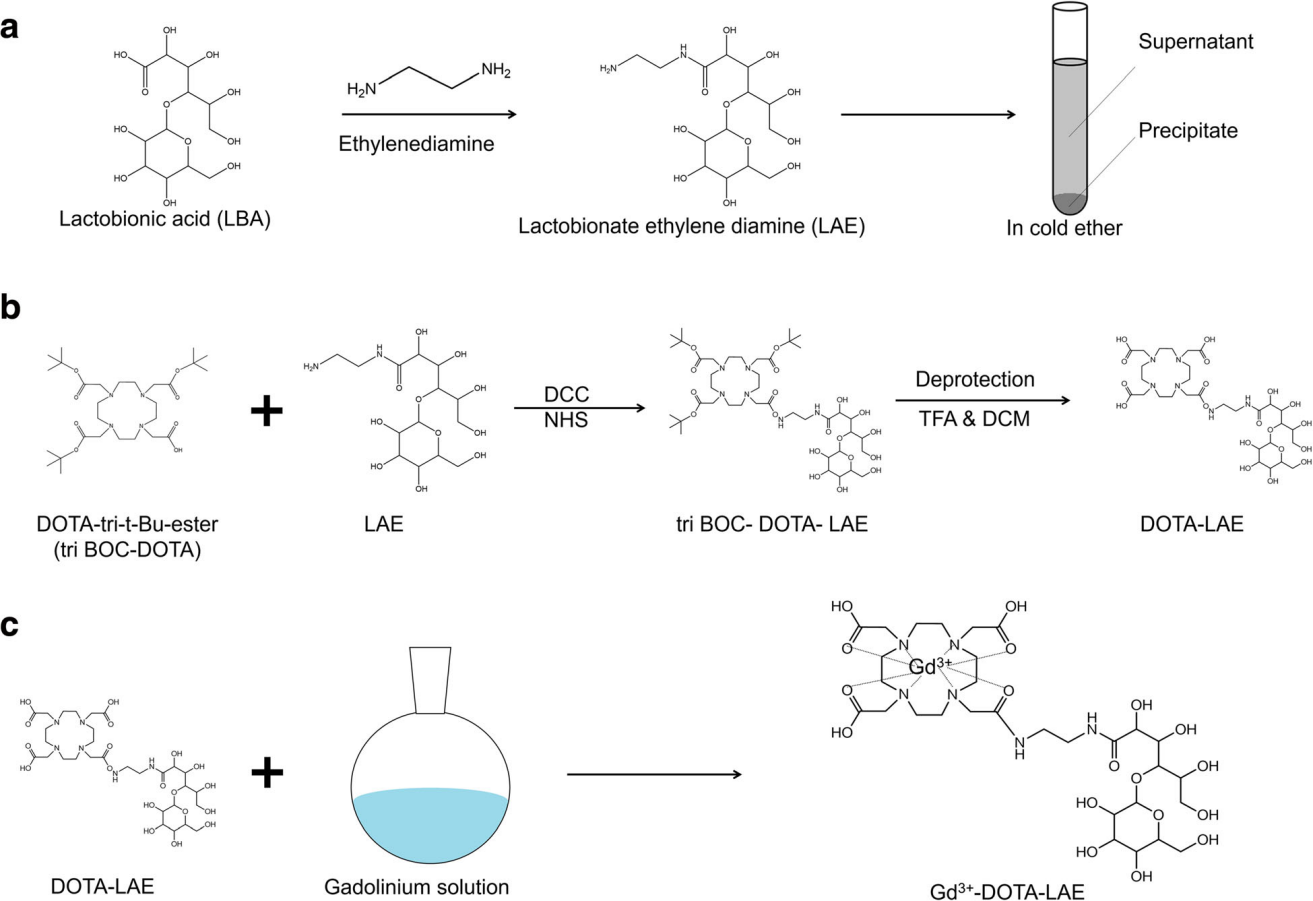
Schematic illustration of the synthetic procedures of Gd-DOTA-LAE. **a** Synthesis process of LAE. **b** Conjugation of p-DOTA with LAE and BOC-deprotection of p-DOTA-LAE using TFA. **c** Grafting of the gadolinium (III) to DOTA-LAE

**Fig. 2:**
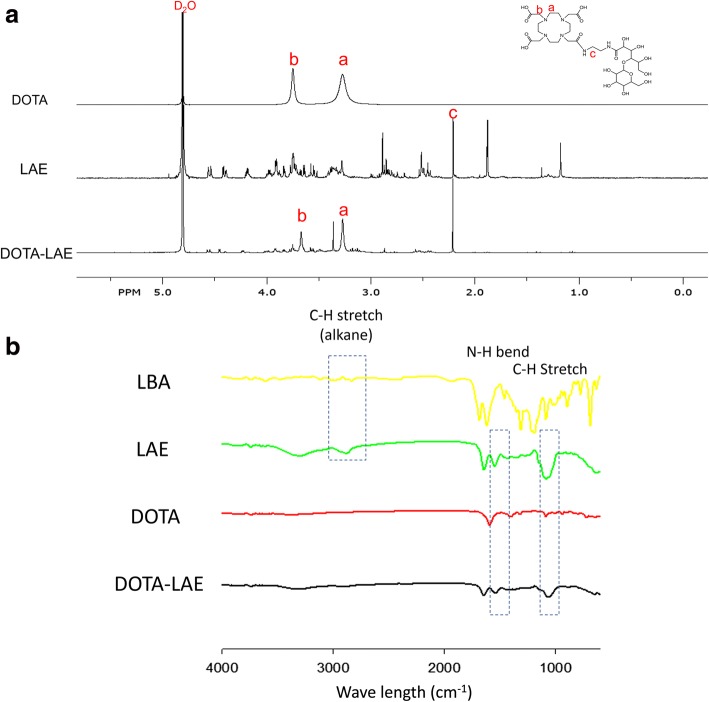
**a** ^1^H NMR spectrum of DOTA, LAE, and Gd-DOTA-LAE. **b** FT-IR spectra of LBA, LAE, DOTA and DOTA-LAE

To synthesize Gd-DOTA-LAE (Fig. 1); carboxyl group of lactobionic acid was modified through the reaction with ethylenediamine to introduce primary amine on lactobionic acid (Fig. 1a) and p-DOTA was introduced to LAE. Tert-butyloxycarbonyl protecting group was remove by reaction with TFA (Fig. 1b). ^1^H NMR spectrum showed hydrogen peaks of DOTA at δ 3.67 ppm (2H,s, CH_2_-N), δ 3.27(4H,s, CH_2_-CH_2_-C) and hydrogen peak of LAE at δ 2.21(4H,m, =CH_2_-CH_2_-N) as shown in Fig. 2a. In addition, FT-IR spectroscopic analysis showed that DOTA was successfully modified to LAE (Fig. 2b). Gd^3+^ was chelated with DOTA-LAE (Fig. 1c). Inductively coupled plasma atomic emission spectroscopy (ICP-MS) was conducted to quantify the contents of Gd^3+^ in Gd-DOTA-LAE (Gd^3+^ contents; 16.0%).

### Comparison of in vitro paramagnetic properties between Gd-DOTA-LAE and Gadovist

**Fig. 3:**
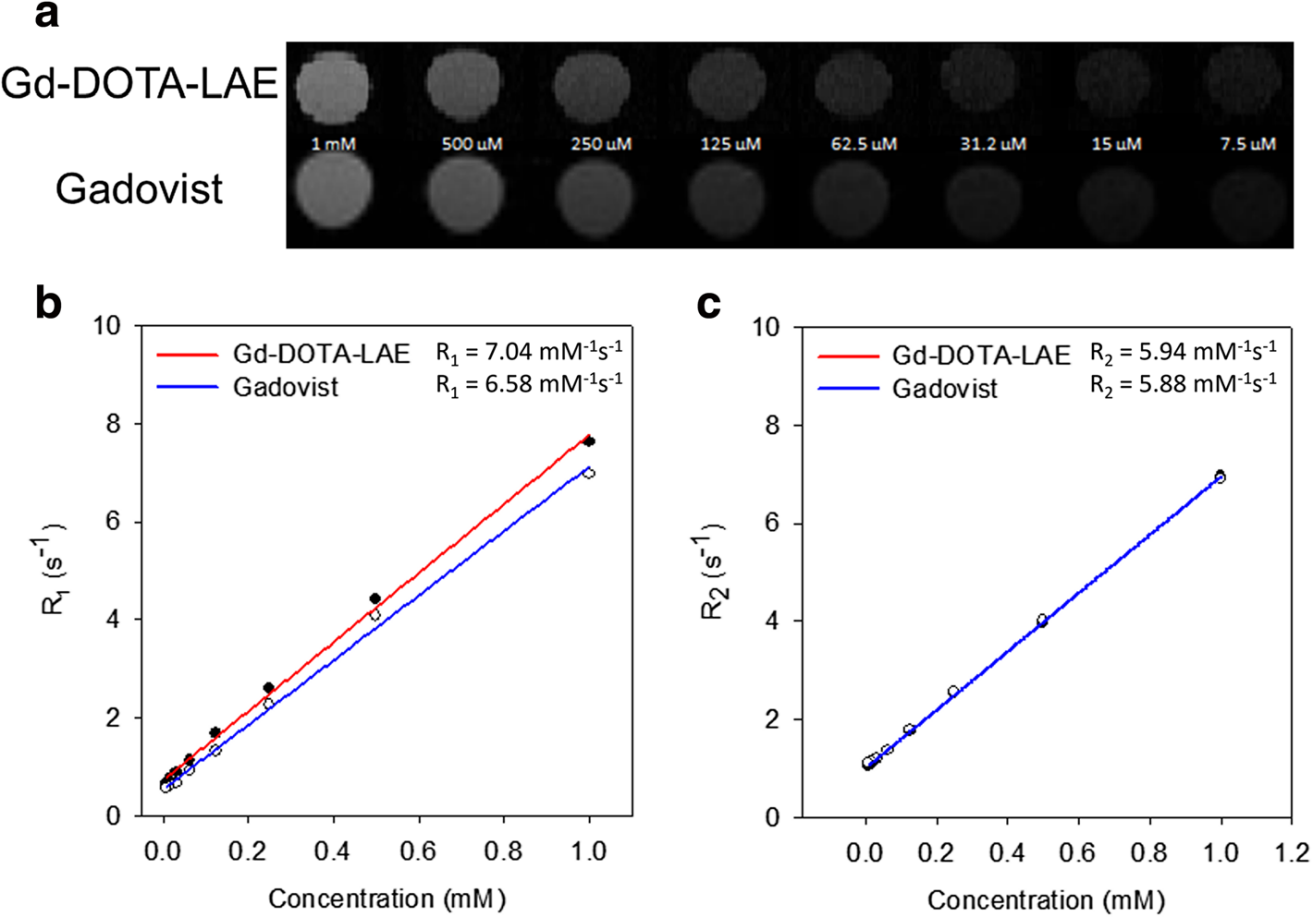
**a** Phantom images of Gd-DOTA-LAE and Gadovist. **b** R_1_ curves of Gd-DOTA-LAE, and Gadovist. **c** R_2_ curves of Gd-DOTA-LAE and Gadovist

Comparison of in vitro paramagnetic properties between Gd-DOTA-LAE and Gadovist was estimated using 4.7 MR scanner. Gadovist was used for comparison study as a macrocyclic chelate-based CA. For CA, R1 and R2 relaxivities of compound are major factor to indicate the contrast efficacy of T1 and T2-weighted CAs. For T1-weighted CAs, shortening T1 relaxation time is important to increase the contrast effect. Therefore, T1-weighted CAs have high R_1_ ratio and low R_2_/R_1_ ratio. On the other hand, T2-weighted CAs have high R2 ratio and relatively high R2/R1 ratio compared to T1-weighted CAs. As shown in Fig. 3a, contrast intensities of phantom images were obtained at various Gd^3+^ ion concentration. GD-DOTA-LAE showed similar contrast intensity with Gadovist (Fig. 3a) and Gd-DOTA-LAE showed the relatively high R_1_ value and similar R_2_ value compare to Gadovist (Fig. 3b & c). In addition, R_2_/R_1_ ratio of Gd-DOTA-LAE (0.84) is relatively lower than that of Gadovist (0.89). These results indicate that Gd-DOTA-LAE may be used for potential CA for T1-weighted MR imaging.

### Cytotoxicity studies of Gd-DOTA-LAE

**Fig. 4:**
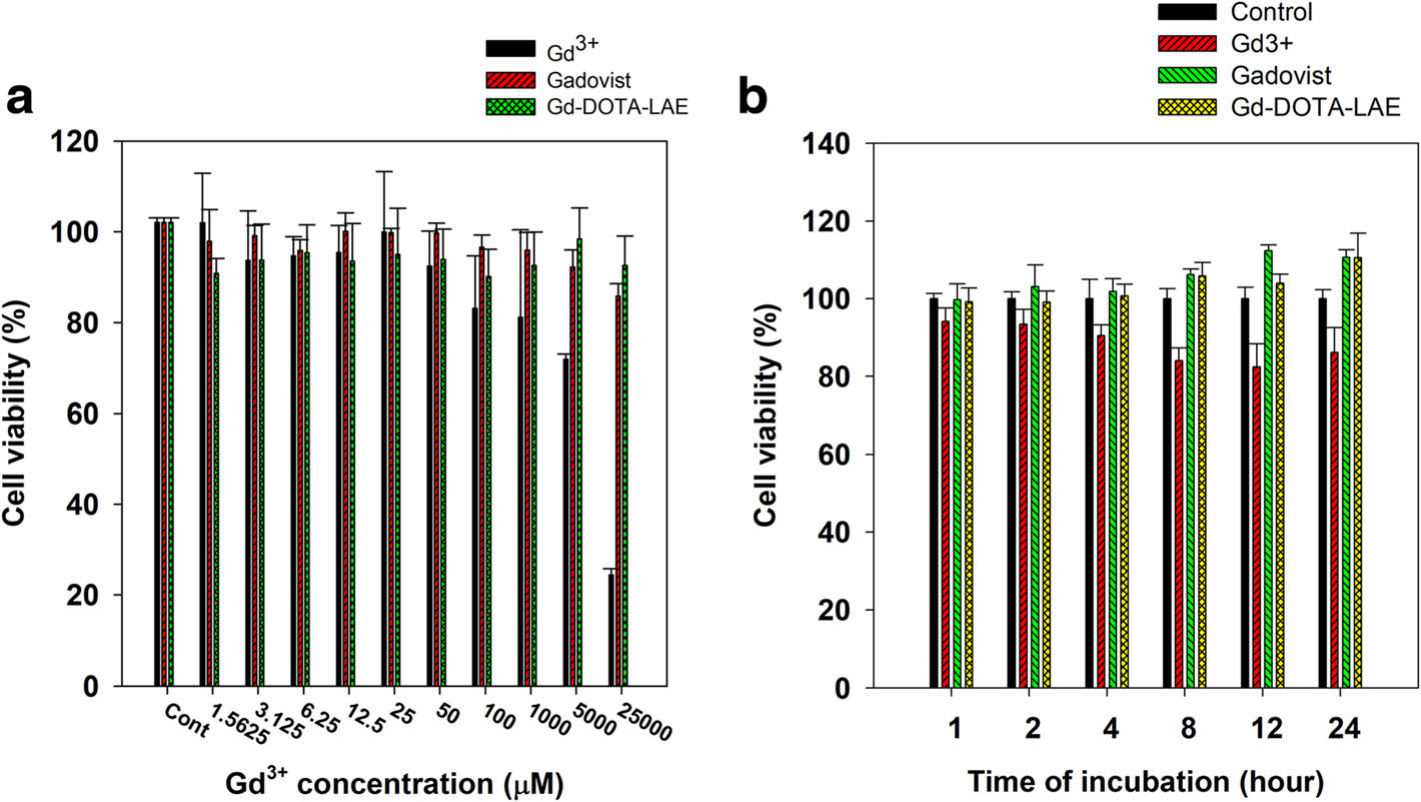
The cell viability of Gd^3+^ in Chang liver cell using MTT assay. **a** change of cell viability in dose-dependent manner. **b** change of cell viability in time-dependent manner (0.1 mmol/L of Gd^3+^)

Gadolinium, as a free ion, is causing serious disease known as nephrogenic systemic fibrosis (NSF) to patients with low kidney function [21]. Therefore, we evaluated the cytotoxicity of Gd-DOTA-LAE, free Gd^3+^ ion and Gadovist on chang liver cell line via MTT assay. Gd-DOTA-LAE and Gadovist showed no serious toxic effect at high dose. Whereas, free Gd^3+^ ion showed a serious toxic effect at high dose. In addition, Gd-DOTA-LAE and Gadovist showed no serious toxic effect in time-dependent manner. We supposed that DOTA-LAE strongly bind to Gd^3+^ ions and minimizing cell interaction with Gd^3+^. Thereby DOTA-LAE did not show the cytotoxicity at high concentration or long periods of time, whereas, free Gd^3+^ showed high cytotoxicity (Fig. 4).

### Concentration of free gadolinium ion and bounded gadolinium

**Fig. 5:**
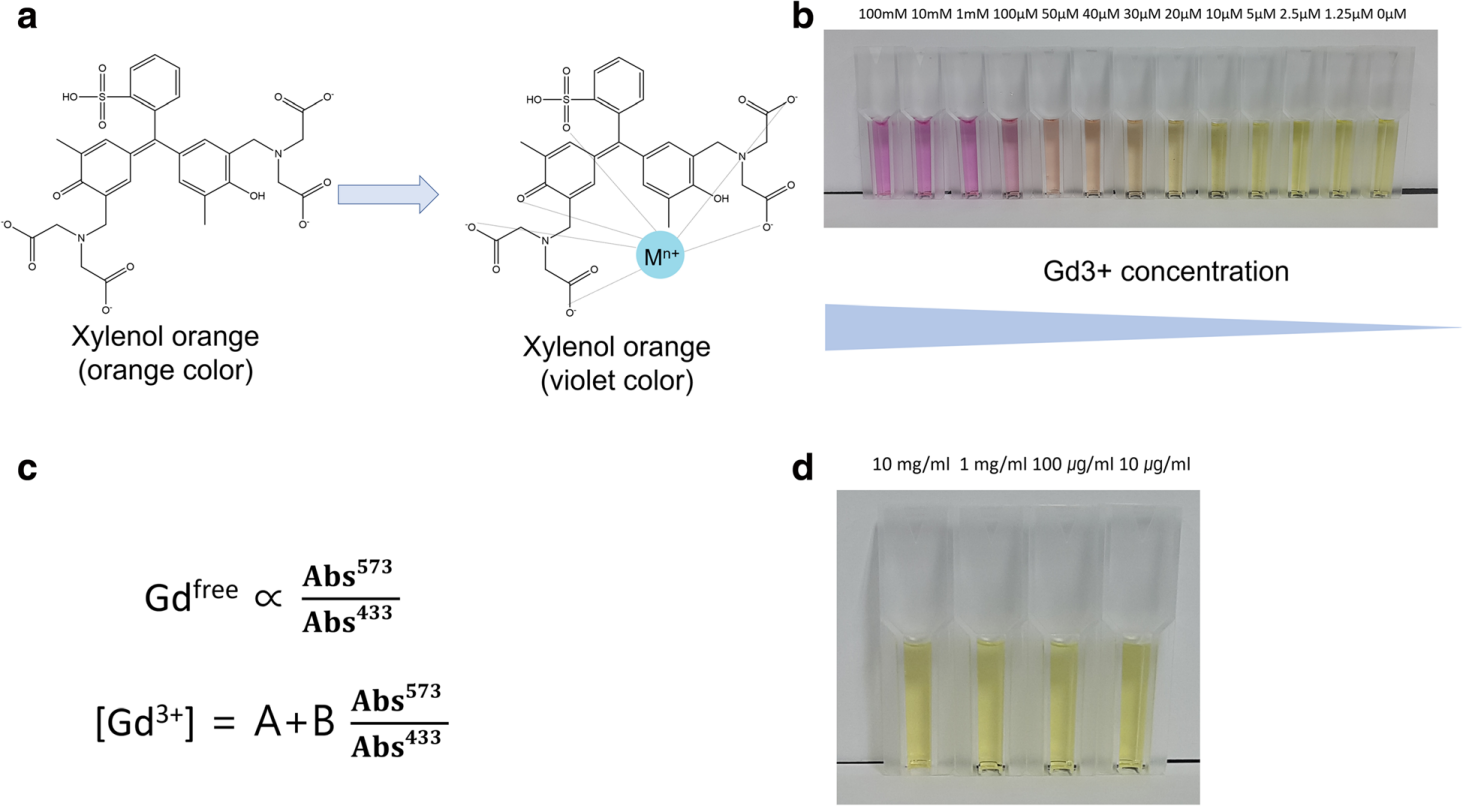
**a** Chemical structure of xylenol orange with or without metal ion. **b** Xylenol orange solution in the presence of Gd3+ ion at various concentrations. **c** Formula for free ion determination. **d** Xylenol orange solution in the presence of Gd-DOTA-LAE

Content of free gadolinium is determined using xylenol orange method (Fig. 5a & b). The ratio of absorbance at 573 nm and 433 nm is proportional to the free Gd^3+^ concentration (Fig. 5c). In 1 g of Gd-DOTA-LAE, 7 μg of free gadolinium (0.007%) contents were determined using this method (Fig. 5d). Xylenol assay and ICP-MS results indicated that most of Gd^3+^ ions are tightly chelated with DOTA-LAE and free Gd^3+^ ions were successfully removed by cation exchange column.

### In vivo MR imaging

**Fig. 6:**
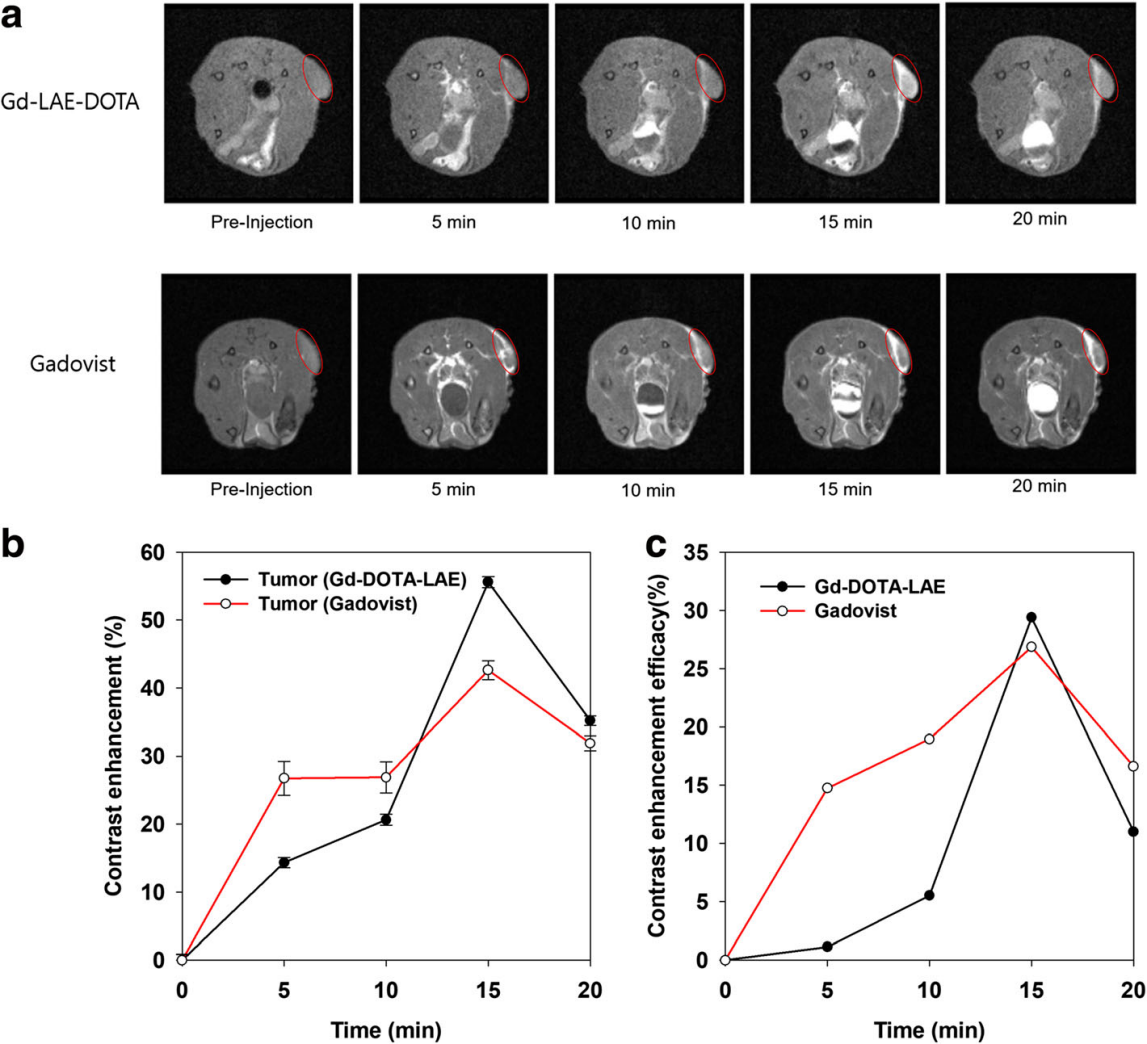
**a** Time-course in vivo T1-weighted MR images of HCT 116 tumor bearing mice after injection of Gd-DOTA-LAE (0.1 mmol/kg) and Gadovist (0.1 mmol/kg). **b** Contrast enhancement of normal tissue and tumor tissue. (contrast intensity post-injection/contrast intensity pre-injection × 100). **c** Contrast enhancement efficacy of Gd-DOTA-LAE and Gadovist in tumor tissue compared to normal tissue. [%, (Tumor post-injection/Tumor pre-injection) × 100 - (normal tissue post-injection/normal tissue pre-injection) × 100]

To evaluate the cancer diagnosis efficacy, 100 μl of Gd-DOTA-LAE (0.1 mmol/kg) and Gadovist (0.1 mmol/kg) were injected into the lateral tail vein of tumor bearing male BALB/c mice and MR image was obtained by 4.7 T animal MR scanner (Fig. 6a). The contrast enhancement of region of interest (ROI) was calculated with the following equation. Contrast enhancement = (ROI _post-injection_/ROI _pre-injection_) × 100. In the case of Gd-DOTA-LAE, normal region showed 20% enhanced T1 contrast within 20 min, the tumor tissue showed 53% enhanced T1 contrast effect within 15 min (Fig. 6b). In the case of Gadovist, normal region showed 20% enhanced T1 contrast within 10 min, the tumor tissue showed 42% enhanced T1 contrast effect within 15 min (Fig. 6b). The contrast enhancement of tumor tissue compared to normal tissue was calculated by following equation. Contrast enhancement efficacy = [%, (Tumor _post-injection_/Tumor _pre-injection_) × 100- (normal tissue _post-injection_/normal tissue _pre-injection_) × 100]. The efficacies of Gd-DOTA-LAE and Gadovist were reached to 29 and 26% at 15 min post injection, respectively (Fig. 6b). Specific accumulation of Gd-DOTA-LAE was observed in cancer region. Therefore, Gd-DOTA-LAE can be used as a CA for cancer diagnosis.

## Discussion

For several decades, various attempts have been applied for accurate diagnosis of cancer. Magnetic resonance imaging is one of the most significant technologies for diagnosis. MR imaging technology helps surgeon make an accurate diagnosis and surgery. In this study, we designed DOTA conjugated lactobionic acid as a tumor diagnosis CA. Gd-DOTA-LAE and Gadovist showed 29 and 26% enhanced contrast intensity in tumor tissues compared to normal tissues within 20 min post injection, respectively. These enhanced contrast effects are explained by the water proton exchange rate. As is known to all, T1-weighted contrast effect is obtained by shortening the spin-lattice relaxation time of water protons around Gd^3+^. We used a lactobionic acid as biocompatible organic acid conjugated with DOTA. In addition, LBA has many hydroxyl groups which improve the water proton density around Gd^3+^ ion by hydrogen bonding, thus it provides the enhanced water proton exchange rate. Therefore, we obtained an enhanced contrast intensity with Gd-DOTA-LAE. Furthermore, there have been reports of nephrogenic systemic fibrosis (NSF) associated with the use of linear chelator-based CA in the past decade. For this reason, there are increasing demands for macrocyclic chelator-based CAs. In this respect, Gd-DOTA-LAE could be used for T1-weighted MR CA in clinical application.

## Conclusion

In conclusion, this study aims to investigate new macrocyclic chelator-based CA. This CA was synthesized DOTA with primary amine modified lactobionic acid using DCC-NHS coupling reaction. In vitro paramagnetic properties showed relatively enhanced T1 contrast effect compared to conventional macrocyclic CA.

In addition, Gd-DOTA-LAE showed 29% enhanced contrast intensity at tumor sites compared to normal tissues within 20 min post injection. These results support that Gd-DOTA-LAE can be used for clinical application for MR imaging.

## Abbreviations

CA: Contrast agent
DOTA: 1,4,7,10-Tetraazacyclododecane-1,4,7,10-tetraacetate
EMA: European Medicine Agency
Gd^3+^: Gadolinium^3+^
LBA: Lactobionic acid
MRI: Magnetic resonance imaging
NSF: Nephrogenic systemic fibrosis
T1: Longitudinal relaxation time
T2: Transverse relaxation time
tri BOC-DOTA: Tri-tert-butyl 1,4,7,10-Tetraazacyclododecane-1,4,7,10-tetraacetate

## References

[1] Jaffer FA, Weissleder R (2005). Molecular imaging in the clinical arena. JAMA.

[2] Koh D-M, Cook GJ, Husband JE (2003). New horizons in oncologic imaging. N Engl J Med.

[3] Caravan P, Ellison JJ, McMurry TJ, Lauffer RB (1999). Gadolinium (III) chelates as MRI contrast agents: structure, dynamics, and applications. Chem Rev.

[4] Li Y, Beija M, Laurent S, Elst LV, Muller RN, Duong HT, Lowe AB, Davis TP, Boyer C (2012). Macromolecular ligands for gadolinium MRI contrast agents. Macromolecules.

[5] Lim J, Turkbey B, Bernardo M, Bryant LH, Garzoni M, Pavan GM, Nakajima T, Choyke PL, Simanek EE, Kobayashi H (2012). Gadolinium MRI contrast agents based on triazine dendrimers: relaxivity and in vivo pharmacokinetics. Bioconjug Chem.

[6] Raymond KN, Pierre VC (2005). Next generation, high relaxivity gadolinium MRI agents. Bioconjug Chem.

[7] Hooker JM, Datta A, Botta M, Raymond KN, Francis MB (2007). Magnetic resonance contrast agents from viral capsid shells: a comparison of exterior and interior cargo strategies. Nano Lett.

[8] Song Y, Xu X, MacRenaris KW, Zhang XQ, Mirkin CA, Meade TJ (2009). Multimodal gadolinium-enriched DNA–gold nanoparticle conjugates for cellular imaging. Angew Chem Int Ed.

[9] Ward K, Aletras A, Balaban RS (2000). A new class of contrast agents for MRI based on proton chemical exchange dependent saturation transfer (CEST). J Magn Reson.

[10] Werner EJ, Datta A, Jocher CJ, Raymond KN (2008). High-relaxivity MRI contrast agents: where coordination chemistry meets medical imaging. Angew Chem Int Ed.

[11] Chen W, Vucic E, Leupold E, Mulder WJ, Cormode DP, Briley-Saebo KC, Barazza A, Fisher EA, Dathe M, Fayad ZA (2008). Incorporation of an apoE-derived lipopeptide in high-density lipoprotein MRI contrast agents for enhanced imaging of macrophages in atherosclerosis. Contrast Media Mol Imaging.

[12] Corot C, Robert P, Lancelot E, Prigent P, Ballet S, Guilbert I, Raynaud JS, Raynal I, Port M (2008). Tumor imaging using P866, a high-relaxivity gadolinium chelate designed for folate receptor targeting. Magn Reson Med.

[13] Kim KS, Park W, Hu J, Bae YH, Na K (2014). A cancer-recognizable MRI contrast agents using pH-responsive polymeric micelle. Biomaterials.

[14] Konda SD, Aref M, Wang S, Brechbiel M, Wiener EC (2001). Specific targeting of folate–dendrimer MRI contrast agents to the high affinity folate receptor expressed in ovarian tumor xenografts. MAGMA.

[15] Shu C-Y, Ma X-Y, Zhang J-F, Corwin FD, Sim JH, Zhang E-Y, Dorn HC, Gibson HW, Fatouros PP, Wang C-R (2008). Conjugation of a water-soluble gadolinium endohedral fulleride with an antibody as a magnetic resonance imaging contrast agent. Bioconjug Chem.

[16] Yim H, Yang S-G, Jeon YS, Park IS, Kim M, Lee DH, Bae YH, Na K (2011). The performance of gadolinium diethylene triamine pentaacetate-pullulan hepatocyte-specific T1 contrast agent for MRI. Biomaterials.

[17] Datta A, Raymond KN (2009). Gd−hydroxypyridinone (HOPO)-based high-relaxivity magnetic resonance imaging (MRI) contrast agents. Acc Chem Res.

[18] Pierre VC, Botta M, Raymond KN (2005). Dendrimeric gadolinium chelate with fast water exchange and high relaxivity at high magnetic field strength. J Am Chem Soc.

[19] Grobner T (2006). Gadolinium–a specific trigger for the development of nephrogenic fibrosing dermopathy and nephrogenic systemic fibrosis?. Nephrol Dial Transplant.

[20] Kuo PH, Kanal E, Abu-Alfa AK, Cowper SE (2007). Gadolinium-based MR contrast agents and nephrogenic systemic fibrosis. Radiology.

[21] Thomson LK, Thomson PC, Kingsmore DB, Blessing K, Daly CD, Cowper SE, Roditi GH (2015). Diagnosing nephrogenic systemic fibrosis in the post-FDA restriction era. J Magn Reson Imaging.

[22] Daram SR, Cortese CM, Bastani B (2005). Nephrogenic fibrosing dermopathy/nephrogenic systemic fibrosis: report of a new case with literature review. Am J Kidney Dis.

[23] Thomsen HS, Morcos SK, Almén T, Bellin M-F, Bertolotto M, Bongartz G, Clement O, Leander P, Heinz-Peer G, Reimer P (2013). Nephrogenic systemic fibrosis and gadolinium-based contrast media: updated ESUR contrast medium safety committee guidelines. Eur Radiol.

[24] Grogna M, Cloots R, Luxen A, Jérôme C, Desreux J-F, Detrembleur C (2011). Design and synthesis of novel DOTA (Gd 3+)–polymer conjugates as potential MRI contrast agents. J Mater Chem.

[25] Barge A, Cravotto G, Gianolio E, Fedeli F (2006). How to determine free Gd and free ligand in solution of Gd chelates. A technical note. Contrast Media Mol Imaging.

